# In-Plane Elastic Properties of 3D-Printed Graded Hierarchical Hexagonal Honeycombs

**DOI:** 10.3390/polym16060859

**Published:** 2024-03-21

**Authors:** Yong Tao, Ruochao Zhao, Jun Shi, De Zhou, Yanqun Han

**Affiliations:** 1 School of Civil Engineering, Central South University, Changsha 410075, China; tao-yong@csu.edu.cn (Y.T.); zhaoruochao@csu.edu.cn (R.Z.); csushijun@csu.edu.cn (J.S.); 210026@csu.edu.cn (D.Z.); 2 Hunan Tieyuan Civil Engineering Testing Co., Ltd., Changsha 410075, China

**Keywords:** graded hierarchical honeycomb, theoretical modeling, finite element analysis, additive manufacturing techniques, in-plane elastic properties

## Abstract

In this study, the graded hierarchical hexagonal honeycomb (GHHH) integrating gradient design and hierarchical design was fabricated using the 3D-printing technique, and its in-plane elastic properties were investigated theoretically, experimentally, and numerically. Theoretical solutions were developed based on the Euler beam theory to predict the effective elastic modulus and Poisson’s ratio of GHHH, and theoretical values were in good agreement with the experimental and numerical results. The effect of gradient design and hierarchical design on the in-plane elastic properties of GHHH was also analyzed and compared. Results showed that the hierarchical design has a more significant effect on Poisson’s ratio and adjusting the internal forces of GHHH compared with the gradient design. In addition, it was found that GHHH exhibited higher stiffness compared with regular hexagonal honeycomb (RHH), graded hexagonal honeycomb (GHH), and vertex-based hierarchical hexagonal honeycomb (VHHH) under the constraint of the same relative density, respectively. Specifically, the effective elastic modulus of GHHH can be enhanced by 119.82% compared to that of RHH. This research will help to reveal the effect of integrating hierarchical design and gradient design on the in-plane elastic properties of honeycombs.

## 1. Introduction

Honeycombs are of immense interest in multifunctional applications because of their excellent specific stiffness and strength [1,2]. Consequently, in the past decades, many studies have been carried out to explore the specific stiffness and strength of honeycombs with different cross-sections such as square [3,4], hexagon [5], and other polygons [6]. However, the performance of these honeycombs is not always satisfactory and usually requires improvement [7]. Therefore, the study of honeycombs with superior performance has become a crucial topic, and many new types of honeycombs have been proposed [8]. With the development of additive manufacturing technologies, the preparation of new honeycombs with complex geometry has become increasingly easy [9]. Among them, gradient honeycombs and hierarchical honeycombs are gaining increasing attention due to their flexible design [10], and gradient design and hierarchical design can effectively improve the performance of honeycombs without increasing their weight [11].

The concept of gradient design is a widely employed strategy in the design of honeycombs [12], and it has been proven through theoretical [13], numerical [14], and experimental [15] studies that the mechanical performance of honeycombs can be significantly affected by gradient design. Based on a non-uniform design method mapped by density-based topology optimization, Zou et al. [16] designed a novel non-uniform honeycomb. It was found that the in-plane and out-of-plane specific energy absorption of the non-uniform honeycomb is, respectively, 29.1% and 19.8% higher than those of regular hexagonal honeycombs. Wu et al. [17] explored the effect of gradient arrangement on the cross-circular honeycomb. The results demonstrated that the dynamic response characteristics of the cross-circular honeycomb with different impact velocities can be effectively controlled by graded design. Based on the concept of the multi-directional graded design, Li et al. [18] proposed the modularized honeycomb and found that the modularized honeycomb possesses higher strength and stronger energy absorption capacity compared with uniform honeycombs. Sahu and Sreekanth [19] investigated an anisotropic gradient honeycomb structure through cyclic compression and free vibration tests. The results demonstrated that compared with length and thickness gradient honeycomb and uniform honeycomb, the hybrid gradient honeycomb possessed the highest damping ability.

The concept of hierarchical design is the other strategy to improve the stiffness, strength, toughness, and energy absorption capacity of honeycombs [20]. Two approaches are commonly utilized to obtain hierarchical honeycombs [21]. One approach involves replacing each vertex of a regular honeycomb with a smaller polygon [22], while the other approach involves replacing the solid cell walls of a regular honeycomb with smaller cellular structures [23]. According to the above two approaches, hierarchical honeycombs can be primarily categorized as vertex-based hierarchical honeycombs and edge-based hierarchical honeycombs. For example, by replacing every three-edge vertex of a regular hexagonal honeycomb with a smaller hexagon, Ajdari et al. [24] proposed a vertex-based hierarchical hexagonal honeycomb. It was found that the stiffness of hierarchical honeycombs of first and second order are 2.0 and 3.5 times that of the regular honeycomb with the same relative density. Hong et al. [25] explored the quasi-static and dynamic compression of multi-level hierarchical honeycomb and single-level hierarchical honeycomb by experimental methods. The results demonstrated that compared with single-level hierarchical honeycomb, multi-level hierarchical honeycomb possesses higher collapse strength and better energy absorption properties. By replacing each solid cell wall of a regular square honeycomb (RSH) with different numbers of smaller square substructures, Tao et al. [26] proposed the square hierarchical honeycomb (SHH). It was shown that the compressive strength, specific energy absorption, and crush force efficiency of the SHH are much greater than those of the RSH. As has been discussed above, both hierarchical design approaches can enhance the mechanical properties of honeycombs [27]. Hence, for better mechanical properties, the hybrid hierarchical square honeycomb (HHSH) combining the geometric features of both edge- and vertex-based hierarchy was investigated by Tao et al. [28]. The results demonstrated that compared with RSH and edge-based hierarchical square honeycomb, the HHSH provided the most excellent crushing performance.

The studies mentioned above are mainly focused on gradient design or hierarchical design. To obtain superior mechanical performance, by integrating hierarchical and gradient designs, a few novel honeycombs are proposed and their mechanical performance is explored [29]. The results indicate that the strategy of integrating gradient design and hierarchical design can further improve the mechanical performance of honeycomb compared with gradient design and hierarchical design [30]. Inspired by the natural honeycomb structure, a novel hierarchical diamond honeycomb with variable cell wall thickness was designed by Tao et al. [31]. It was found that the out-of-plane shear modulus of the novel hierarchical diamond honeycomb with variable cell wall thickness was related to the material distribution. Taylar et al. [32] explored the functional grading in hierarchical honeycomb and found that Young’s modulus is significantly impacted by the aspect ratio of the super-structure. By varying the fractal parameter of the uniform self-similar honeycomb for each layer, Liu et al. [33] proposed a functionally graded fractal honeycomb. It was found that, under low impact velocity impact, the absorbed energy of graded fractal honeycomb can be improved by up to 89% compared with that of traditional hexagonal honeycomb. In order to obtain the more stable deformation of the honeycomb, Liu et al. [34] introduced a structural gradient into continuous woven glass fiber-reinforced hierarchical thermoplastic composite honeycomb graded structures. It was found that compared with regular configurations, core components of staggered configurations can absorb more energy.

As discussed above, it can be seen that graded hierarchical honeycombs integrating gradient design and hierarchical design greatly increase the designability of the honeycomb, and it is expected to achieve various excellent performances such as stiffness, strength, and energy absorption while achieving a lightweight material. Therefore, it has high academic research value and huge application prospects. However, at present, compared with regular honeycomb, graded honeycomb, and hierarchical honeycomb, research on graded hierarchical honeycomb is scarce. The in-plane elastic properties of honeycombs integrating gradient and vertex-based hierarchy have not been reported. By incorporating vertex-based hierarchy and gradient into the hexagonal honeycomb, the novel graded hierarchical hexagonal honeycomb (GHHH) was proposed in our previous study [35]. The results illustrated that the plateau stress and specific energy absorption of GHHH were much superior to those of regular hexagonal honeycomb, vertex-based hierarchical hexagonal honeycomb, and graded hexagonal honeycomb. To further reveal the effect of integrating gradient and hierarchical designs into honeycombs, the in-plane effective elastic properties of GHHH are investigated by theoretical prediction, experimental test, and numerical simulation. The sections of this study are arranged as follows. The geometric configuration of GHHH is described in Section 2. The macroscopic in-plane linear elastic properties of GHHH are studied theoretically based on Euler beam theory in Section 3. Subsequently, in Section 4, the details of the fabrication and testing of GHHHs are presented, and the numerical model of GHHH is validated by the experimental and theoretical results. Next, the simulation results on effective elastic modulus and Poisson’s ratio of GHHH are presented and compared with the theoretical results in Section 5. Finally, Section 6 concludes the main findings of this study.

## 2. Geometric Description of Graded Hierarchical Hexagonal Honeycomb

**Figure 1:**
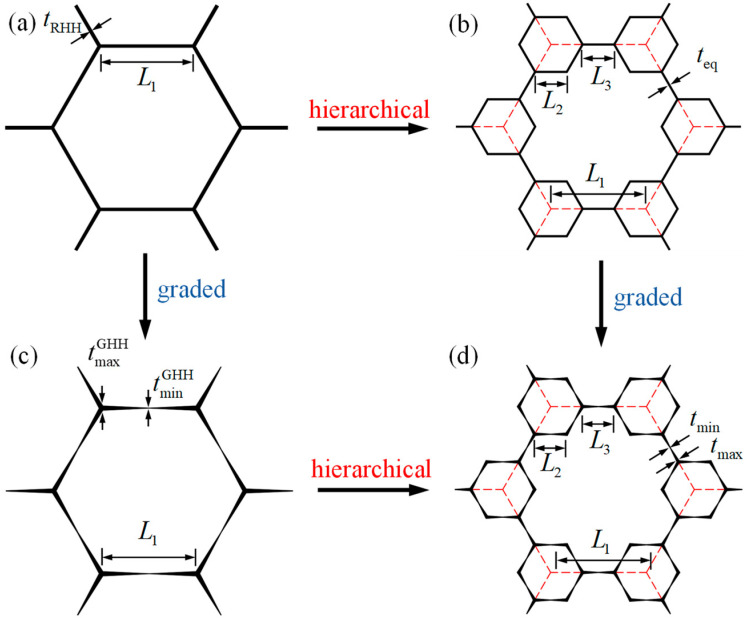
The geometric configurations of (**a**) RHH, (**b**) VHHH, (**c**) GHH, and (**d**) GHHH.

**Figure 2:**
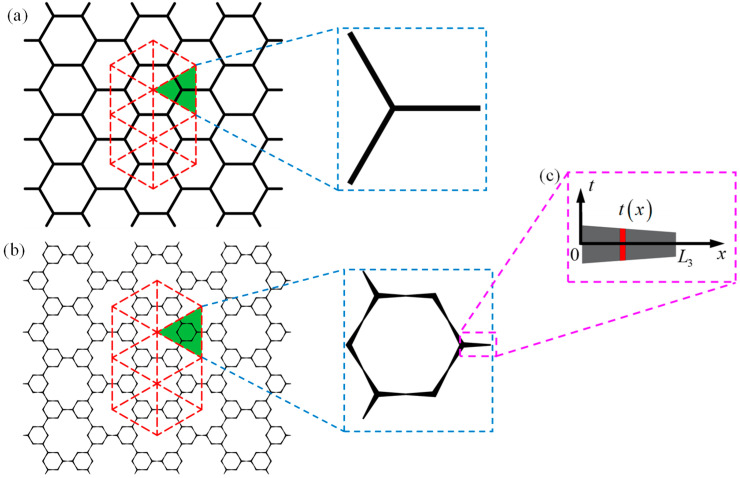
Schematic of the unit cells of (**a**) RHH, (**b**) GHHH, and (**c**) schematic of the edge of GHHH in the local coordinate system.

The hexagon is the most renowned cell topology of honeycombs [31]. Figure 1a shows a regular hexagonal honeycomb (RHH), and it has been widely reported that gradient design and hierarchical design can significantly improve the mechanical properties of RHH [10]. Hence, to enhance the performance of honeycombs, the vertex-based hierarchical hexagonal honeycomb (VHHH, Figure 1b) is constructed by replacing every vertex of the RHH, and the graded hexagonal honeycomb (GHH, Figure 1c) is developed by introducing cell wall thickness variation into the RHH. Furthermore, based on our previous work [35], the graded hierarchical hexagonal honeycomb (GHHH, Figure 1d) investigated in this study can be obtained by introducing cell wall thickness variation into the VHHH. The detailed geometric construction process of GHHH is schematized in Figure 1, and the representative unit cells of RHH and GHHH are shown in Figure 2a,b, respectively.

The hierarchical structural parameter λ is defined to describe the geometric configuration of VHHH and GHHH, which can be calculated as:

(1) $$\lambda =\frac{2L_{2}}{L_{1}}$$

where $L_{1}$ is the side length of RHH and GHH, and $L_{2}$ is the side length of the smaller hexagon for VHHH and GHHH, as shown in Figure 1. The constraint of 0≤λ≤1 should be met to avoid geometric overlap. To better describe the material distribution of GHHH, the gradient parameter k is defined as:

(2) $$k=\frac{t_{\max}-t_{\min}}{t_{eq}}$$

where $t_{\max}$ and $t_{\min}$ are the maximum and minimum cell wall thickness of GHHH, respectively, and $t_{eq}$ is the equivalent cell wall thickness, which can be calculated by $t_{eq}=\left(t_{\max}+t_{\min}\right)/2$. As illustrated in Figure 2b, the cell wall thickness of each half side length of GHHH can be expressed by:

(3) $$t\left(x\right)=t_{\max}-\left(t_{\max}-t_{\min}\right)\frac{2x}{L_{i}},\;0\leq x\leq \frac{L_{i}}{2},\;i=2,\;3$$

where x is the distance from the origin of the local coordinate system, as shown in Figure 2c, and the side length of $L_{3}$ can be calculated by $L_{3}=L_{1}-2L_{2}$. Similarly, the gradient parameter for GHH can be defined as:

(4) $$k=\frac{t_{\max}^{\mathrm{GHH}}-t_{\min}^{\mathrm{GHH}}}{t_{\mathrm{RHH}}}$$

where $t_{\max}^{\mathrm{GHH}}$ and $t_{\min}^{\mathrm{GHH}}$ are the maximum and minimum cell wall thickness of GHH, respectively, and $t_{\mathrm{RHH}}$ is the cell wall thickness of the GHH with k=0. And the cell wall thickness of GHH can be calculated by:

(5) $$t\left(x\right)=t_{\max}^{\mathrm{GHH}}-\left(t_{\max}^{\mathrm{GHH}}-t_{\min}^{\mathrm{GHH}}\right)\frac{2x}{L_{1}},\;0\leq x\leq \frac{L_{1}}{2}$$

The relative density $\bar{\rho }$ of the honeycomb is the most important parameter to determine its properties, and it can be defined by the ratio of the density of the honeycomb to the density of the constituent material. For GHHH and VHHH, their relative density can be expressed by [35]:

(6) $$\bar{\rho }=\frac{t_{eq}}{\sqrt{3}L_{1}}\left(1+\lambda \right)$$

It should be pointed out that the relative density of GHHH with an equivalent cell wall thickness $t_{eq}$ and VHHH with a cell wall thickness $t_{eq}$ is the same. In addition, when k=0, GHHH degrades to VHHH. And when λ=0, RHH and GHH can be obtained from VHHH and GHHH, respectively. Hence, the relative density of RHH and GHH can be calculated by:

(7) $$\bar{\rho }=\frac{t_{\mathrm{RHH}}}{\sqrt{3}L_{1}}$$

Similarly, when k=0, GHH degrades to RHH. In addition, RHH also can be obtained from GHHH when λ=0 and k=0.

## 3. Theory of Effective Elastic Modulus and Poisson’s Ratio

**Figure 3:**
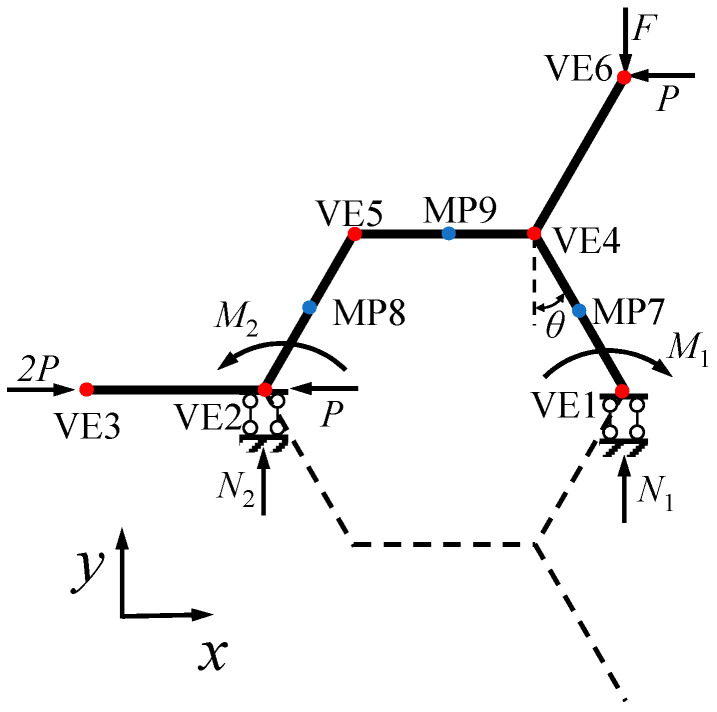
Mechanical model of the subassembly of GHHH.

In this section, Euler beam theory is adopted to investigate the in-plane uniaxial deformation of GHHH made of an isotropic elastic material with elastic modulus $E_{s}$. Since GHHH owns threefold symmetry, its in-plane elastic properties are isotropic and do not depend on the direction [24,36]. Hence, the macroscopic in-plane linear elastic properties of GHHH can be characterized by two independent elastic parameters, namely effective elastic modulus $E^{*}$ and Poisson’s ratio $\upsilon^{*}$ [24,36]. When a far field *y*-direction stress $\sigma_{yy}=-2F/3L_{1}h$ applied to the hexagonal honeycomb with an expanding angle of $120^{\circ }$, where h is the out-of-plane depth of the hexagonal honeycomb, the theoretical model of the hexagonal honeycomb can be equivalent to the mechanical model of its subassembly [24]. The mechanical model of the subassembly of GHHH is shown in Figure 3. It should be pointed out that all horizontal forces presented in Figure 3 are not actual loads, they are the dummy forces based on the assumption of Castigliano’s second theorem. Therefore, the horizontal force P=0 is finally adopted. When the GHHH is loaded in the *y*-direction, the cell walls of GHHH not only carry bending moments but also axial and shear forces. Since the ratio of the cell wall thickness to effective cell length is small, the shear deformation of GHHH can be negligible relative to the bending deflection [36]. However, the cell wall thickness of GHHH is variable. It leads to the discovery that the axial deformation of GHHH cannot be negligible and should be considered in the analysis [36]. In addition, as the deformation of GHHH is very small, the beam-column and geometric non-linearity can be disregarded. According to the equilibrium equation, one can have:

(8) 2P−P−P=0

(9) $$N_{1}+N_{2}-F=0$$

(10) $$N_{1}L_{2}\left(1+2\sin\theta \right)+P\left(L_{2}+\frac{L_{3}}{2}\right)\cos\theta +M_{2}-F\left[L_{2}+\left(L_{2}+L_{3}\right)\sin\theta \right]-M_{1}=0$$

where $N_{1}$ and $M_{1}$ are the reaction force and bending moment at point VE1, respectively; $N_{2}$ and $M_{2}$ are the reaction force and bending moment at point VE2, respectively; and θ are the angles as presented in Figure 3. Based on Castigliano’s second theorem, the strain energy of the unit cell of GHHH can be calculated as a sum over all the edges:

(11) $$U\left(F,P,N_{1},M_{1}\right)=\int \frac{M^{2}\left(x\right)}{2E_{s}I}dx+\int \frac{N^{2}\left(x\right)}{2E_{s}A_{s}}dx$$

where M(x) and N(x) are the bending moment and axial force at a distance of x from the origin of the local coordinate system; $I=ht^{3}\left(x\right)/12$ is the moment of inertia of cell walls; and $A_{s}=ht\left(x\right)$ is the cross-sectional area of the cell wall of GHHH. Because of symmetry boundary conditions, there is zero y displacement and zero rotation at point VE1. Therefore, on the basis of Castigliano’s second theorem, the partial derivative of strain energy over $N_{1}$ and $M_{1}$ is 0. It can be expressed by:

(12) $$\frac{\partial U}{\partial M_{1}}=0$$

(13) $$\frac{\partial U}{\partial N_{1}}=0$$

The $N_{1}$, $M_{1}$, $N_{2}$, and $M_{2}$ can be obtained by substituting Equations (8)–(12) into Equation (13). At point VE6, the x and y displacements can be calculated by:

(14) $$\delta_{x}=\frac{\partial U}{\partial P}$$

(15) $$\delta_{y}=\frac{\partial U}{\partial F}$$

Hence, the effective elastic modulus of GHHH is defined as the ratio of average stress to average strain ($\varepsilon_{y}=-4\delta_{y}/\sqrt{3}L_{1}$) in the *y*-direction, and it can be calculated by:

(16) $$E^{*}=\frac{\sigma_{yy}}{\varepsilon_{y}}=\frac{\sqrt{3}F}{6\delta_{y}h}$$

Poisson’s ratio of GHHH is defined as the opposite of the ratio of average strain ($\varepsilon_{x}$) in the *x*-direction to average strain in the *y*-direction, and it can be calculated by:

(17) $$\upsilon^{*}=-\frac{\varepsilon_{x}}{\varepsilon_{y}}=\frac{\delta_{x}}{\sqrt{3}\delta_{y}}$$

## 4. Experimental and Numerical Methods

### 4.1. Experimental Test

**Figure 4:**
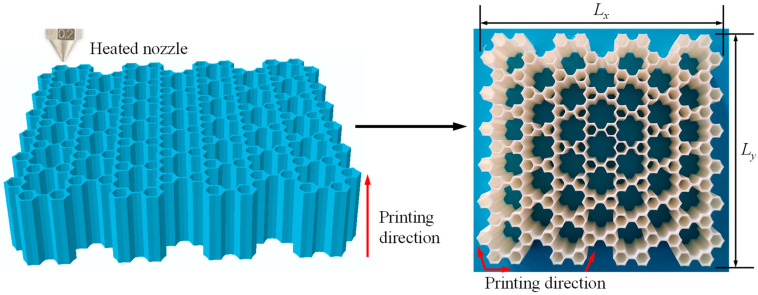
The preparation process of the GHHH specimens.

Since the geometric dimensions and internal structures of GHHH integrate hierarchical design and gradient design, the geometry of GHHH is very complex, and the GHHH is difficult to manufacture using traditional manufacturing methods. Hence, the fused deposited modeling (FDM) method is adopted in this study to fabricate the GHHH. All the specimens in this study were printed using a Raise 3D Pro3 printer (Nantong, China). Compared with other additive manufacturing techniques like selective laser melting (SLM), the Raise 3D Pro 3 printer with FDM using various filaments is versatile in terms of printability [37], which is beneficial for the design, fabrication, and optimization of different GHHHs. The constituent material adopted for 3D printing was PLA with desirable mechanical properties, and the printing quality of PLA was consistent and acceptable. Hence, the PLA is widely used in additive manufacturing techniques [38,39]. According to the requirements of the Raise 3D Pro3 printer, before transferring the geometric model of the GHHH to the Raise 3D Pro3 printer, a CAD model of the GHHH established using SolidWorks 2023 software should be partitioned into slices using slicing software (ideaMaker 4.3.3). To ensure the accuracy and quality of the GHHH printed by the Raise 3D Pro3 printer, the printing parameters were set based on the characteristics of PLA and the printer. The diameter of the heated nozzle was 0.2 mm, and the temperature of the heated nozzle was 215 °C. The infill density was 100%, while the printing speed was 50 mm/s. The single layer thickness and bottom layer thickness were 0.2 mm and 0.3 mm, respectively. All specimens were printed at room temperature. To ensure that the bottom layer of the GHHH could be easily separated from the build platform after printing and firmly attached to the build platform without warping, the build platform was kept at 55 °C. The printing direction was along the out-of-plane direction of the GHHH. In the *x-y* plane, the printing direction was along the cell wall of the GHHH. The preparation process of the GHHH printed by the Raise 3D Pro3 printer is shown in Figure 4. As shown in Figure 4, $L_{x}$ is the total length of GHHH in the *x* direction, while $L_{y}$ is the total length of GHHH in the *y* direction. In addition, the geometric parameters of each GHHH specimen are tabulated in Table 1. The relative density in Table 1 is obtained according to Equation (6).

**Figure 5:**
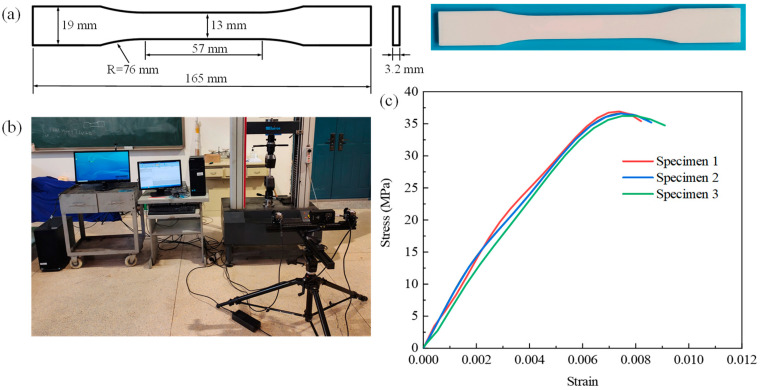
(**a**) The geometry of the dog-bone shaped tensile specimens, (**b**) experimental setup, and (**c**) stress–strain curves of PLA.

To obtain the mechanical properties of the solid cell walls of GHHH, three tensile dog-bone specimens were also printed, and quasi-static tensile tests were performed on them based on the ASTM D638-22 standard [40]. The tensile velocity was kept at 0.5 mm/min. A digital image correlation technique (DIC) was adopted in this study to measure the strain of dog-bone specimens. The geometric dimensions of dog-bone specimens and the experimental setup are shown in Figure 5. According to the test results, stress–strain curves of PLA are plotted in Figure 5c, and the basic mechanical properties of PLA were obtained as follows: density $\rho_{s}=1200\;\mathrm{kg}/m^{3}$, Young’s modulus $E_{s}=2100\;\mathrm{MPa}$, and Poisson’s ratio υ=0.35.

**Figure 6:**
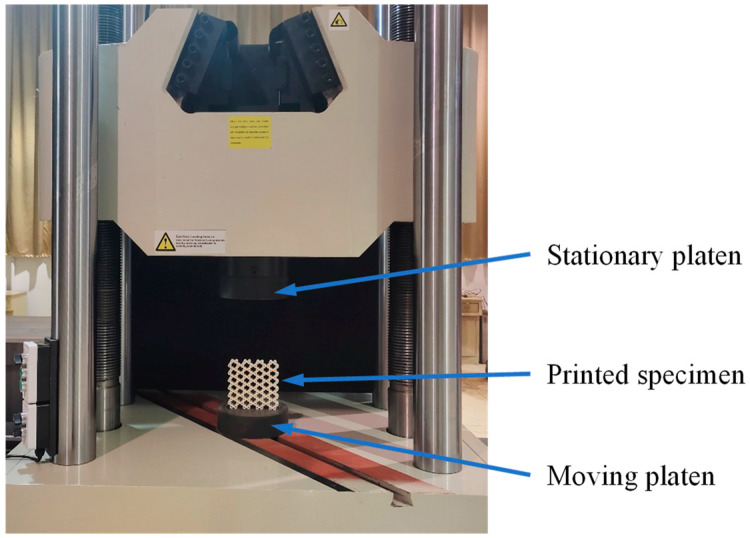
In-plane quasi-static compressive test setup for GHHH specimen.

The experimental setup of the in-plane quasi-static compression test on the GHHH is shown in Figure 6. It can be found from Figure 6 that GHHH was sandwiched between the top platen and bottom platen. In-plane compressive tests of GHHH specimens were conducted using a hydraulic universal testing machine with a load cell of 2000 kN. The top platen was stationary, while the bottom platen was moved upwards with a constant velocity of 0.5 mm/min along the *y*-direction to load the honeycomb specimen. The effective elastic modulus of GHHH was obtained from the slope of the stress–strain curve at the early stage of the experiment (the strain of GHHH should be less than 1.5%) [24].

### 4.2. Finite Element Modeling

**Figure 7:**
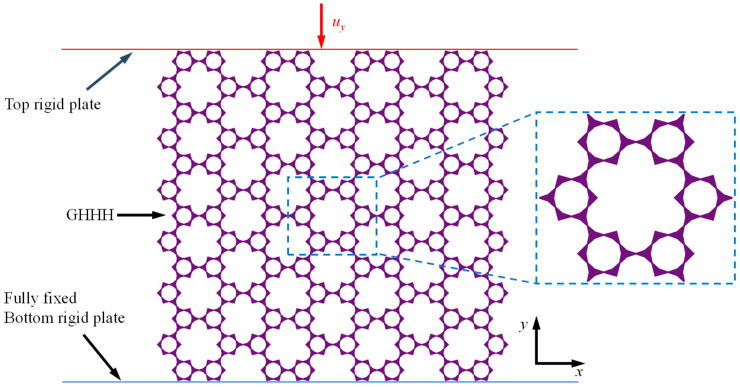
Finite element model of GHHH subjected to in-plane loading.

In order to study the elastic properties of GHHH, the in-plane loading process of GHHH was simulated using the finite element software ABAQUS/Standard 6.14-4. As illustrated in Figure 7, GHHH was placed between two analytical rigid plates. The ends of GHHH were connected to the stationary bottom rigid plate by tie constraint. The stationary bottom rigid plate was fully fixed and all directions of the top plate were constrained except for the y direction. A constant displacement was loaded onto the top plate along the y direction. To simulate the possible contact, the surface-to-surface contact model was adopted between the GHHH and two plates, and the general contact was set between the surfaces of GHHH. For simplicity, the two types of contacts were frictionless [21]. Since the elastic deformation of out-of-plane is sufficiently small, the out-of-plane deformation effects are ignored and GHHH is meshed using the CPS4R element. CPS4R is the 4-node bilinear plane stress quadrilateral element and possesses reduced integration with hourglass control, which can better simulate the in-plane loading process of GHHH and has high computational efficiency [41]. In addition, to ensure the numerical results are accurate, a mesh sensitivity analysis was conducted for the finite element model as well. The constituent material of GHHH is PLA, and its mechanical properties are given in Section 4.1. To avoid boundary effects, the strain was calculated within the representative cell of GHHH (blue ellipse in Figure 7). Poisson’s ratio of GHHH can be calculated by the opposite of the ratio of the average strain of the representative cell of GHHH in the *x*-direction to the average strain in the *y*-direction.

In order to determine the best mesh size and eliminate mesh sensitivity, a mesh convergence analysis with different mesh sizes of 0.01 mm, 0.02 mm, 0.04 mm, and 0.08 mm was conducted for the GHHH with k=0.3, λ=0.3, and $\bar{\rho }=0.01$. The result demonstrates that the mesh size of 0.02 mm produced the best convergence as shown in Table 2. The differences in effective elastic modulus between mesh sizes 0.02 mm and 0.01 mm were less than 4.30%. Hence, a mesh size of 0.02 mm was adopted in this study.

### 4.3. Validation of Finite Element Modeling Method

**Figure 8:**
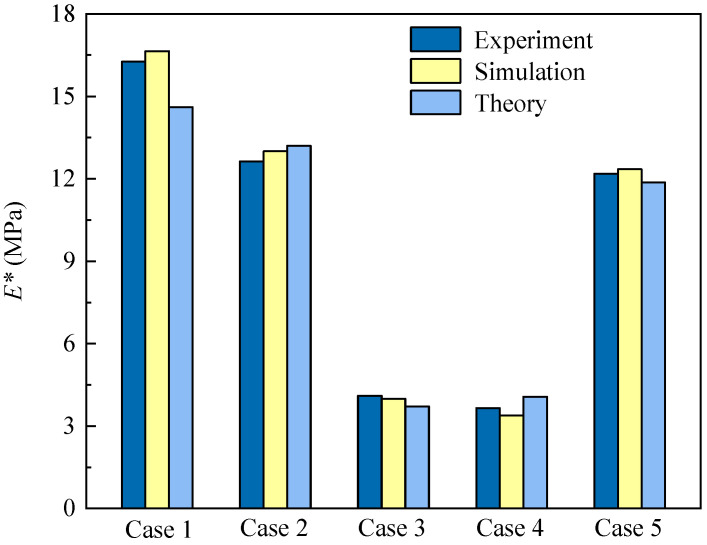
Comparison of experimental, numerical, and theoretical effective elastic modulus.

To validate the accuracy of the finite element modeling method proposed in Section 4.2, the finite element analysis on the GHHH was conducted and compared with the experimental test and theoretical analysis. Figure 8 presents the effective elastic modulus obtained from experiment, simulation, and theory. It can be found that the effective elastic modulus of the finite element model agrees well with that of the experimental and theoretical results. Therefore, the finite element model can well predict the in-plane elastic properties of GHHH.

## 5. Results and Discussion

### 5.1. Effective Elastic Modulus

**Figure 9:**
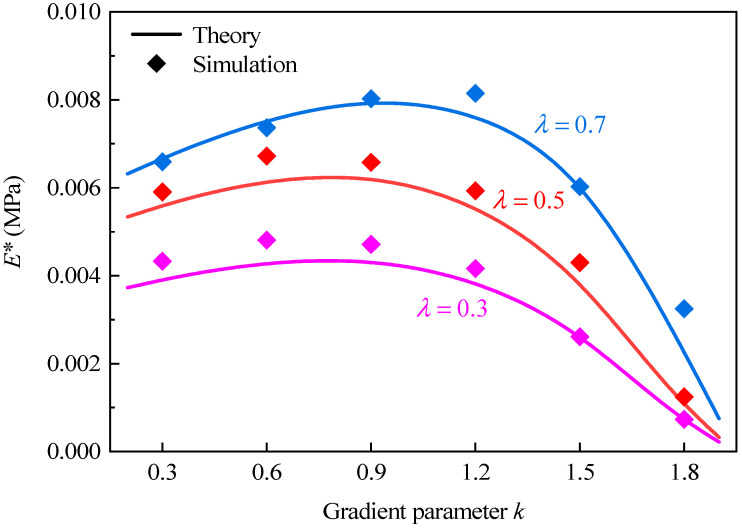
The effective elastic modulus of GHHH versus k when λ=0.3, 0.5, and 0.7.

Figure 9 presents the effective elastic modulus of GHHH versus k when λ=0.3, 0.5, and 0.7. Here, the relative density $\bar{\rho }$ is fixed to 0.01 for comparison, and the gradient parameter k ranges from 0.3 to 1.8 with an interval of 0.3. It can be seen from Figure 9 that with the increase in k, the effective elastic modulus of GHHH increases at first and then decreases. Theoretically, the bending deformation of each cell wall of honeycombs is related to its moment of inertia [42], and the maximum bending occurs at the vertices of the honeycombs when subjected to uniaxial loading [43]. With the increase in k, the cell wall thickness at the vertex $t_{\max}$ increases. Therefore, the moment of inertia at the vertex increases, and the bending deformation of GHHH decreases, resulting in the increase in the effective elastic modulus of GHHH. However, as k further increases, the cell wall thickness $t_{\min}$ in the middle of the cell wall of GHHH decreases sharply. It leads to the moment of inertia in the middle of the cell wall decreasing and the middle area of the cell wall becoming increasingly frail. Hence, the ability of GHHH to resist deformation is weakened and the effective elastic modulus of GHHH decreases.

**Figure 10:**
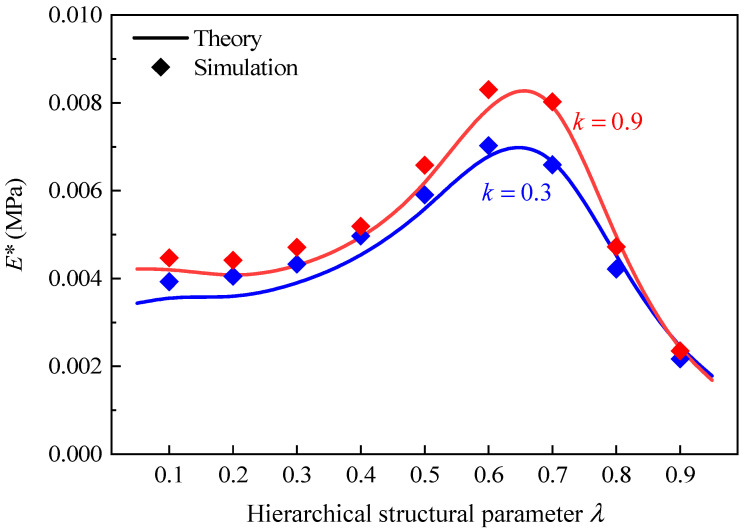
The effective elastic modulus of GHHH versus λ when k=0.3 and 0.9.

In addition, it can also be found from Figure 9 that the decreases in effective elastic modulus of GHHH due to k become more pronounced with the increase in λ. According to Section 2, by substituting Equation (2) into Equation (6), $t_{\min}$ can be calculated as $t_{\min}=\sqrt{3}\left(2-k\right)L_{1}\bar{\rho }/\left(2+2\lambda \right)$. When k, $L_{1}$, and $\bar{\rho }$ remain unchanged, the $t_{\min}$ decreases with the increase in λ, further resulting in a weakening of the ability of GHHH to resist deformation. As a result, compared with the effective elastic modulus of GHHH with λ=0.3, according to the theoretical analysis, the effective elastic modulus of GHHH with λ=0.5 and λ=0.7 is smaller when k=1.8. Furthermore, the variation in the effective elastic modulus of GHHH ($\bar{\rho }=0.01$) with the hierarchical structural parameter λ is shown in Figure 10. The hierarchical structural parameter λ varies from 0.1 to 0.9 with an interval of 0.1. One can observe that the effective elastic modulus of GHHH initially increases and subsequently decreases with the increase in λ. It is well known that the bending deformation of the cell wall of a honeycomb is related to its effective length [42]. With the increase in λ, the effective length of the cell wall of GHHH subjected to bending moments becomes longer. Materials between point VE2 and point VE3 (Figure 3) do not bear bending moments and the length between point VE2 and point VE3 decreases. Hence, with increase in λ, there are more materials to resist bending deformation and thus the effective elastic modulus of GHHH increases. However, with a further increase in λ, since $t_{\min}=\sqrt{3}\left(2-k\right)L_{1}\bar{\rho }/\left(2+2\lambda \right)$, the cell wall thickness $t_{\min}$ in the middle of the cell wall of GHHH gradually decreases. The moment of inertia $I=ht_{\min}^{3}/12$ [24] in the middle of the cell wall of GHHH decreases and the middle area of the cell wall becomes increasingly frail. Therefore, the ability of GHHH to resist deformation is weakened and the effective elastic modulus of GHHH decreases.

**Figure 11:**
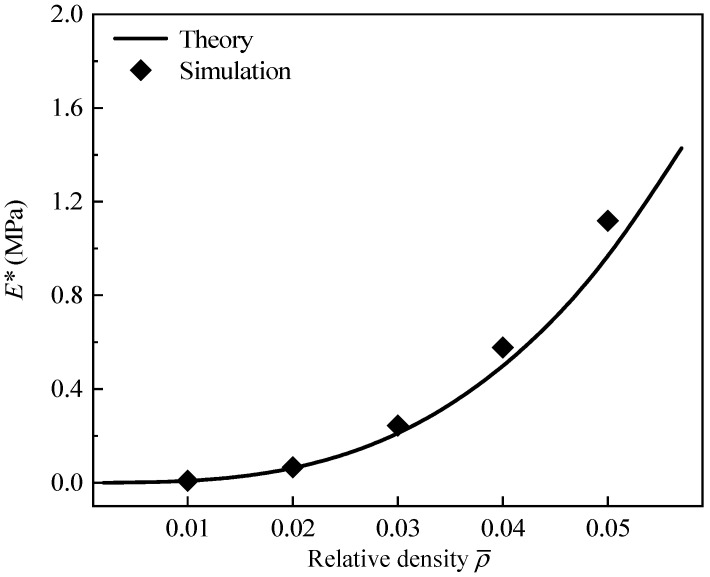
The effective elastic modulus of GHHH versus $\bar{\rho }$ when k=0.9 and λ=0.6.

Furthermore, the effect of relative density on the effective elastic modulus of GHHH is also explored. Figure 11 presents the effective elastic modulus of GHHH versus $\bar{\rho }$ when k=0.9 and λ=0.6. The relative density $\bar{\rho }$ varies from 0.01 to 0.05 with an interval of 0.01. It can be seen from Figure 11 that effective elastic modulus increases with the increase in relative density. It can be attributed to the increase in moment of inertia caused by the increase in relative density.

### 5.2. Poisson’s Ratio

**Figure 12:**
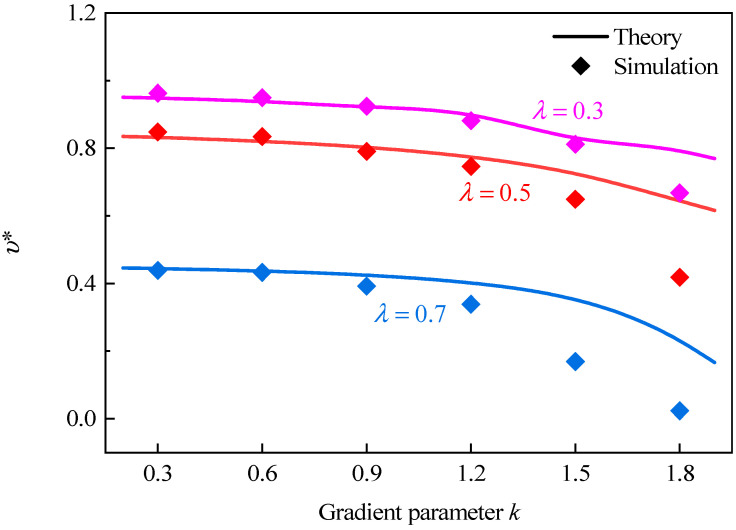
Poisson’s ratio of GHHH versus k when λ=0.3, 0.5, and 0.7.

Figure 12 shows Poisson’s ratio of GHHH with different k when $\bar{\rho }=0.01$. It can be seen that with the increase in the gradient parameter k, Poisson’s ratio of GHHH slowly decreases. Obviously, the theoretical prediction is in good agreement with the numerical simulation when the gradient parameter k and hierarchical structural parameter λ are small. However, with the increases in the gradient parameter k and hierarchical structural parameter λ, the error between theoretical and numerical Poisson’s ratio of GHHH is relatively large. This can be explained as the cell walls of GHHH are simplified to Euler beams. As we all know, the Euler beam theory applies the plane assumption and assumes that Euler beams are composed of many longitudinal fibers. Hence, the ratio of cell wall thickness to side length should be sufficiently small to satisfy the above assumptions [44,45]. Sun et al. [44] suggested that the ratio should be less than 0.25. In a different study, Ding et al. [45] stated that the ratio should be less than 0.2. However, the honeycombs studied in the two papers both have a uniform cell wall thickness. For honeycombs with uniform cell wall thickness, the shear stresses on adjacent cross-sections of the uniform cell wall are identical, resulting in the same degree of warping, and the displacements caused by warping are the same. Therefore, the length of longitudinal fibers of the cell wall of the honeycomb with a uniform cell wall thickness does not change due to cross-sectional warping, and no additional tensile stress is induced. In other words, cross-sectional warping does not alter the tensile stress according to the plane assumption. However, as the cell wall thickness of GHHH is variable, the adjacent cross-sections of GHHH are different. As a result, the shear stresses on adjacent cross-sections are different, leading to varying degrees of warping. Consequently, the length of the longitudinal fibers of cell wall of GHHH undergoes changes, thereby causing additional tensile stresses. This is precisely the factor overlooked by the plane assumption. Hence, for GHHH, the ratio of cell wall thickness to side length should be smaller compared with that of honeycomb with uniform cell wall thickness. Meanwhile, it should be pointed out that the difference between the shear stresses on adjacent cross-sections will become larger with the increase in k, resulting in an increase in error.

**Figure 13:**
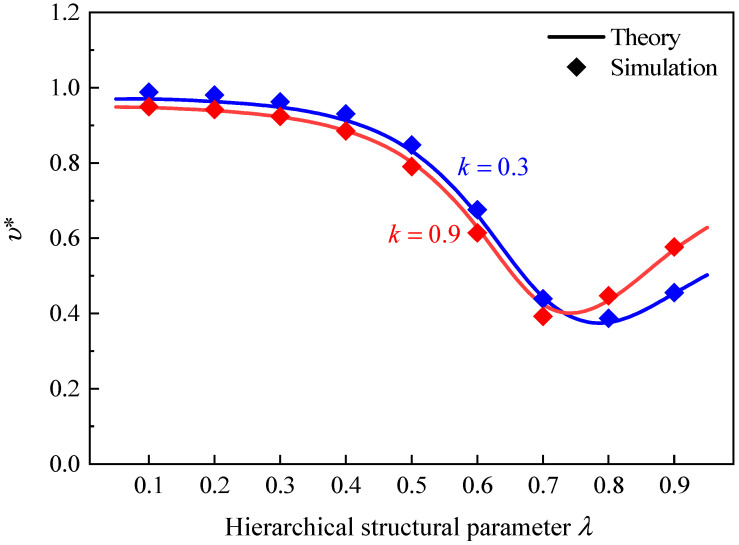
Poisson’s ratio of GHHH versus λ when k=0.3 and 0.9.

**Figure 14:**
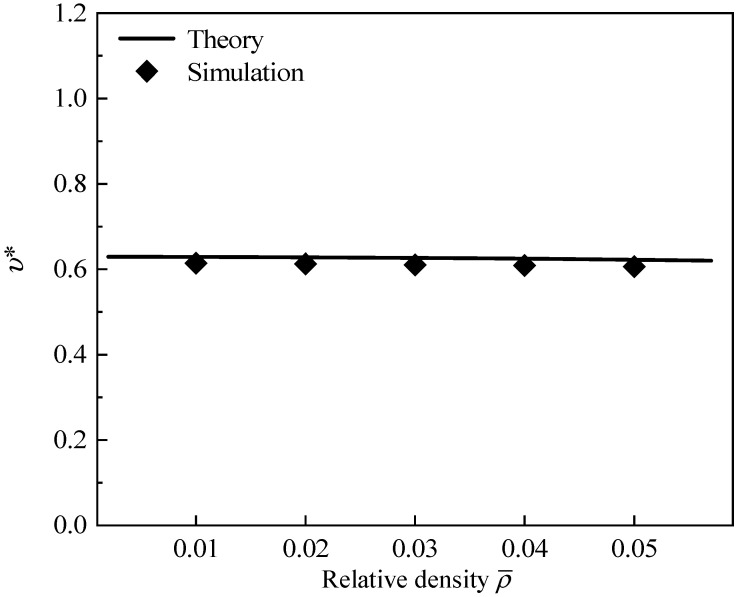
Poisson’s ratio of GHHH versus $\bar{\rho }$ when k=0.9 and λ=0.6.

Figure 13 presents the effect of the hierarchical structural parameter λ on Poisson’s ratio of GHHH with k=0.3 and k=0.9. The relative density $\bar{\rho }$ is set to 0.01. As can be found from Figure 13, as λ increases, Poisson’s ratio first decreases and then increases. Specifically, GHHH with smaller λ has a Poisson’s ratio close to 1.0. With the increase in λ, a more uniform stress distribution and smaller strain may be achieved, leading to a reduction in Poisson’s ratio. With the further increase in λ, according to Section 5.1, the ability of GHHH to resist deformation weakens. This leads to GHHH having larger deformation, resulting in a higher Poisson’s ratio. The variation in Poisson’s ratio of GHHH with the same relative density $\bar{\rho }$ is presented in Figure 14. Note that the k is fixed to 0.9 and λ is fixed to 0.6. As shown in Figure 14, a good consistency is still achieved between the theoretical prediction and numerical simulation. It can be seen from Figure 14 that with the increase in relative density, Poisson’s ratio slightly decreases and remains almost unchanged. This means that the relative density has little effect on Poisson’s ratio of the GHHH.

### 5.3. Effect of Integrating Hierarchical and Gradient Designs

Ajdari et al. [24] and Chuang et al. [42] showed that the effective elastic modulus of hexagonal honeycomb is significantly influenced by hierarchical design and gradient design, respectively. As shown in Section 2, GHHH explored in this study integrates the geometry of both hierarchical design and gradient design. Therefore, GHHH may own outstanding elastic performance. In this section, the effect of integrating hierarchical design and gradient design on the elastic performance of hexagonal honeycombs is investigated through the comparison of the elastic mechanical properties of RHH, VHHH, GHH, and GHHH. In this section, the relative density of all honeycombs is fixed to 0.01.

**Figure 15:**
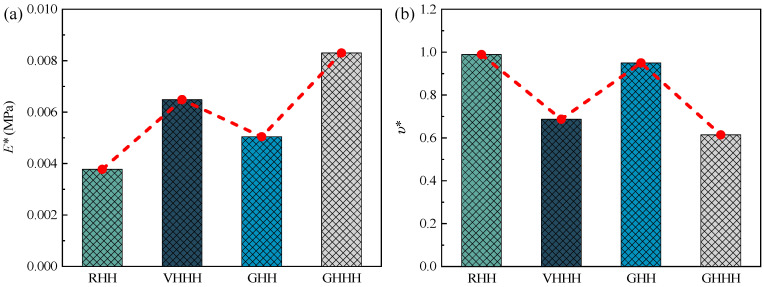
Comparison of (**a**) effective elastic modulus and (**b**) Poisson’s ratio between different honeycomb configurations.

**Figure 16:**
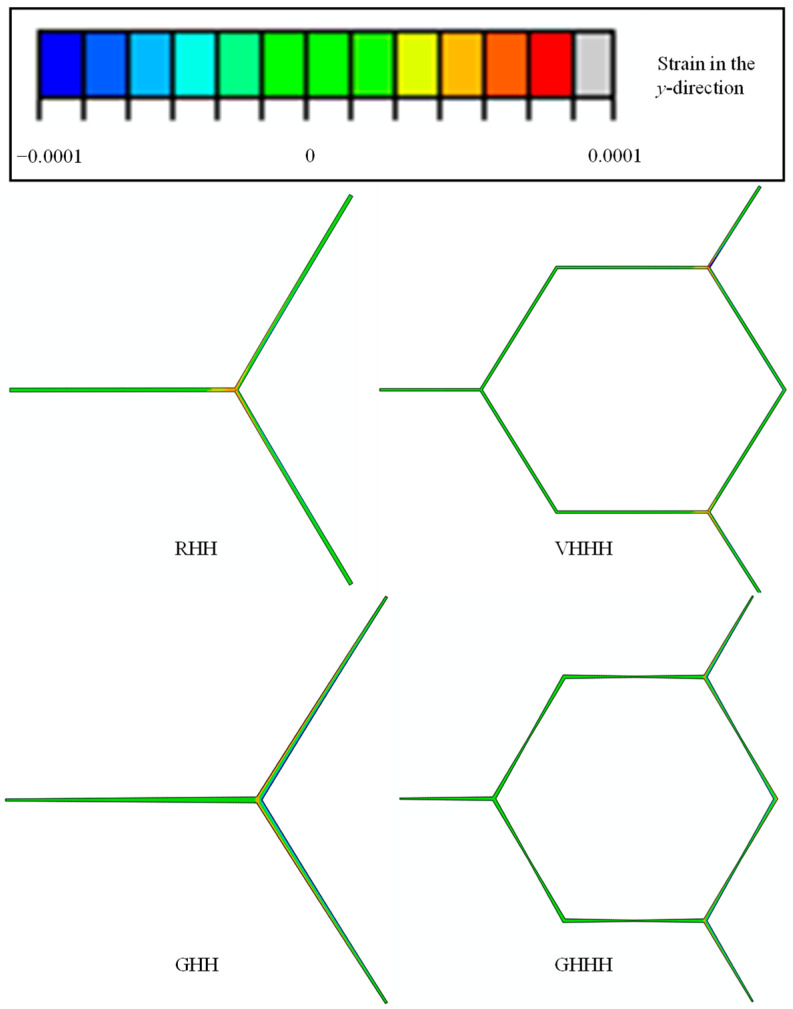
Deformation modes of unit cells of RHH, VHHH, GHH, and GHHH.

As shown in Figure 15, the comparison of effective elastic modulus and Poisson’s ratio between GHHH and its degraded honeycomb (i.e., RHH, VHHH, and GHH) is plotted. Here, the hierarchical structural parameter λ of VHHH and GHHH is set as 0.6, while the gradient parameter k of GHH and GHHH is fixed to 0.9. It can be seen from Figure 15a that the effective elastic modulus of VHHH, GHH, and GHHH are improved by up to 71.69%, 33.51%, and 119.82% compared with that of RHH, respectively. This demonstrates that the GHHH integrating hierarchical design and gradient design can significantly improve the effective elastic modulus compared to the GHH with gradient design and VHHH with hierarchical design if the relative density is fixed. In addition, the deformation modes of unit cells with the same stress of RHH, VHHH, GHH, and GHHH are shown in Figure 16. As illustrated in Figure 16, strains of all honeycombs in the *y*-direction are concentrated at the vertex, which is consistent with theoretical analysis. GHHH not only increases the vertex count by replacing each vertex of RHH with a smaller hexagon but also assigns more material near the vertices by introducing cell wall thickness variation. Hence, compared with RHH, VHHH, and GHH, GHHH possesses more material subjected to resistance to bending moment, and the strains of GHHH in the *y*-direction are smaller. Therefore, GHHH has the highest effective elastic modulus. As shown in Figure 15b, Poisson’s ratios of RHH and GHH are almost the same and both are close to 1.0. This indicates that the gradient design has little effect on Poisson’s ratio of RHH. When hierarchical design is introduced into RHH and GHH, respectively, Poisson’s ratio has significantly decreased. Hence, the hierarchical design has a more significant effect on Poisson’s ratio compared with the gradient design. In addition, Poisson’s ratio of GHHH is 10.57% lower than that of VHHH. This means that gradient design can further tailor Poisson’s ratio of honeycomb on the basis of hierarchical design.

**Figure 17:**
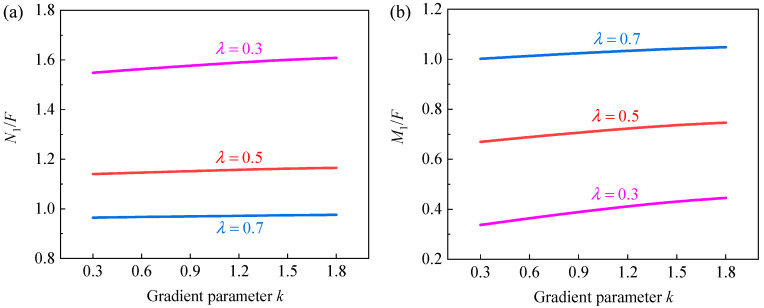
The (**a**) $N_{1}/F$ and (**b**) $M_{1}/F$ versus k when λ=0.3, 0.5, and 0.7.

**Figure 18:**
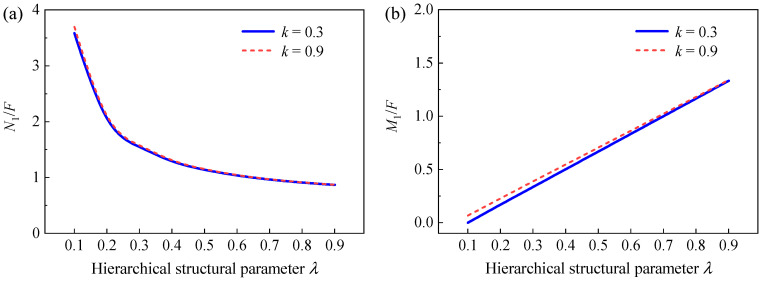
The (**a**) $N_{1}/F$ and (**b**) $M_{1}/F$ versus λ when k=0.3 and 0.9.

To further explore the effect of gradient design and hierarchical design on the in-plane elastic properties of GHHH, Figure 17 and Figure 18 present the effect of k and λ on the ratio of force $N_{1}$ to F and ratio of the bending moment $M_{1}$ to F at point VE1 (Figure 3) of GHHH, respectively, where $N_{1}$ and $M_{1}$ can be obtained from Section 3. As shown in Figure 17, $N_{1}/F$ and $M_{1}/F$ slightly increase with the increase in k. And it can be seen from Figure 18 that with the increase in λ, $N_{1}/F$ rapidly decreases while $M_{1}/F$ experiences a substantial increase. Specifically, $N_{1}/F$ of GHHH with k=0.9 and λ=0.1 is 4.26 times of that of GHHH with k=0.9 and λ=0.9, while $M_{1}/F$ of GHHH with k=0.9 and λ=0.9 is 19.62 times of that of GHHH with k=0.9 and λ=0.1. Hence, one can conclude that gradient design has little effect on the distribution of internal forces in GHHH while hierarchical design can change the internal force distribution of GHHH to improve the elastic performance of GHHH.

## 6. Conclusions

In this study, the in-plane elastic properties of the graded hierarchical hexagonal honeycomb (GHHH) integrating gradient design and hierarchical design were investigated through theoretical analysis, experiment, and numerical simulation. Based on the analysis, the main conclusions are drawn as follows:

(1) Theoretical models to predict the effective elastic modulus and Poisson’s ratio of GHHH were developed based on Euler beam theory, and theoretical results were in good agreement with experimental and numerical results.
(2) The gradient parameter k and hierarchical structural parameter λ have an important effect on the effective elastic modulus and Poisson’s ratio of GHHH. In addition, Poisson’s ratio is less sensitive to the relative density.
(3) The effect of gradient design and hierarchical design on the in-plane elastic properties of GHHH has been analyzed and compared. Compared to gradient design, hierarchical design shows a more pronounced effect on Poisson’s ratio and adjusting the internal forces of GHHH.
(4) The effective elastic modulus of GHHH is higher than that of RHH, VHHH, and GHH when the relative density is fixed. The effective elastic modulus of GHHH can be up to 2.2 times that of RHH. However, Poisson’s ratio of GHHH is lower than that of RHH, VHHH, and GHH.

To sum up, the GHHH explored in this study exhibits excellent stiffness. The strategy of integrating gradient design and hierarchical design in honeycomb provides new possibilities for a more flexible design of honeycomb materials to meet complex actual needs. However, the proposed work focused only on the in-plane elastic properties of GHHH. The crashworthiness performance of GHHH under the in-plane impact will be explored in future studies, and the elastic response under the out-of-plane is currently under study.

## Figures and Tables

**Table 1:** The detailed parameters of the GHHH specimens.

Specimen	$L_{x}$ (mm)	$L_{y}$ (mm)	$L_{1}$ (mm)	*h* (mm)	$t_{eq}$ (mm)	λ	k	$\bar{\rho }$
Case 1	141.32	132.78	12	30	0.8	2/3	0.5	0.1283
Case 2	141.04	132.54	12	30	0.8	2/3	0.25	0.1283
Case 3	136.64	128.73	12	30	0.8	0.3	0.25	0.1001
Case 4	136.99	129.03	12	30	0.8	0.3	1	0.1001
Case 5	142.99	134.22	12	30	0.8	0.8	1	0.1386

**Table 2:** Comparison of effective elastic modulus between finite element models with different element sizes.

Element Size (mm)	Effective Elastic Modulus (10^−4^ MPa)	Error (%)
0.01	45.18	-
0.02	43.28	−4.21
0.04	5.21	−87.95
0.08	0.14	−97.36

## Data Availability

Data are available upon reasonable request.
